# Supplementary material for: Getting More Out of Biomedical Documents with GATE's Full Lifecycle Open Source Text Analytics
Source: PLoS Comput Biol. 2013 Feb 7;9(2):e1002854. doi: 10.1371/journal.pcbi.1002854 (PMC3567135; doi:10.1371/journal.pcbi.1002854)
Supplement: Dataset S1 — GATE software. Dataset S1 bundled with this paper contains a distribution of GATE (or see http://gate.ac.uk/download/). (TGZ) [file pcbi.1002854.s001.tgz › plos-gate/src/gate/creole/annic/apache/lucene/store/package.html]

Binary i/o API, used for all index data.
